# Chronic inflammation promotes proliferation in the prostatic stroma in rats with experimental autoimmune prostatitis: study for a novel method of inducing benign prostatic hyperplasia in a rat model

**DOI:** 10.1007/s00345-020-03090-6

**Published:** 2020-01-21

**Authors:** Mengyang Zhang, Changcheng Luo, Kai Cui, Tao Xiong, Zhong Chen

**Affiliations:** 1 Department of Urology, Tongji Hospital, Tongji Medical College, Huazhong University of Science and Technology, Wuhan, 430030 Hubei China; 2 Institute of Urology, Tongji Hospital, Tongji Medical College, Huazhong University of Science and Technology, Wuhan, 430030 Hubei China

**Keywords:** Animal models, Inflammation, Benign prostatic hyperplasia, Oxidative stress

## Abstract

**Objective:**

Inflammation plays an important role in the development of benign prostatic hyperplasia (BPH). The aim of the present study was to reference the study of the pathological changes in the prostate gland of rats with experimental autoimmune prostatitis (EAP), for the development of experimental models of BPH.

**Methods:**

Experimental autoimmune prostatitis was induced in rats by the intradermal injection of rat prostate antigen with immunoadjuvants. In case of the positive BPH group, BPH was induced by the subcutaneous injection of testosterone propionate. At the end of the 45-day model period, prostate weights were measured, and the histopathological analysis of the prostate glands was performed. The levels of cytokines, TGF-β1/RhoA/ROCK signals, and the oxidative stress status were also examined.

**Results:**

Rats from the EAP group had a higher histological score than those from the control group. Compared to the samples from rats in the hormone-induced group, those from the EAP group showed a more pronounced increase in the size of the stromal compartment; this was characterized by the formation of reactive stroma and the deposition of a greater amount of extracellular matrix (ECM). Significant increases in the numbers of CD3-positive cells and CD68-positive cells, as well as a significant upregulation in the cytokine levels, and an increase in the TGF-β1 levels and activation of RhoA/ROCK signaling, were observed in the samples from rats in the EAP group.

**Conclusion:**

Chronic inflammation can induce BPH in rats via EAP model method. When performing drug experiments on the stroma compartments of BPH, the use of the EAP model is a recommendation of the authors based on this study.

## Introduction

Benign prostate hyperplasia (BPH) is a common benign disease in old men. The incidence of the gross enlargement of the prostate gland has been reported to be 40% in 70-year-old men, and microscopic foci of the prostate gland are present in up to 80% of these men [1]. BPH is a multifactorial disease; androgens, estrogens, stromal–epithelial interactions, growth factors, and neurotransmitters may play a role, either singly or in combination, in the etiology of the hyperplastic process [2]. In recent years, it has been considered that inflammation is not just an occasional concomitant occurrence accompanying BPH, but instead, plays a key role in its etiology [3]. This is manifested by the high incidence of inflammation that has been found in excised BPH samples [4], and the degree of inflammation has shown a significant correlation with the severity of BPH [5-7]. BPH has been hypothesized to be an immune-mediated inflammatory disease, and inflammation may directly contribute to prostate growth [8].

At present, the hormone-induced animal model of BPH is mainly used for BPH-related studies. Indeed, testosterone plays an essential role in the development of BPH. In 1976, Walsh and Wilson [9] first reported the successful use of hormones in castrated dogs to induce a BPH model. Based on their research, the animals currently used as models in many BPH drug therapy experiments are predominantly rats, and the model method is based on large doses of androgen, with castration or no castration, and androgen alone or a combination of estrogen and androgen [10–13]. However, the serum testosterone level of humans decreases with age [14]; hence, large exogenous doses of testosterone contradict the natural evolution of BPH. The hormone-induced models may thus, not fully represent the true nature of BPH.

To expand the scope of the model methods of BPH, other causes of BPH, such as inflammation, should be considered for the induction of BPH models. To mimic the appearance of inflammation with BPH, which is consistent with its natural evolution, we attempted to induce chronic inflammation in rats to investigate the pathological changes of the prostate gland and the related underlying mechanisms.

## Material and methods

### Animals and experimental design

A total of 36 male Sprague–Dawley rats (9 weeks old, 250–300 g) were supplied by the Laboratory Animal Center of the Tongji Medical College, Huazhong University of Science and Technology. The rats were housed in individual cages in a room maintained at 20–26 °C with a relative humidity of 35–75%; they were subjected to an alternating 12-h light–dark cycle (8:00 a.m. and 8:00 p.m.). The animals had free access to food and water. All procedures involving the animals were approved by the Institutional Animal Care and Use Committee of the Tongji Hospital, Tongji Medical College, Huazhong University of Science and Technology (Hubei, China). After 7 days of acclimatization, the rats were randomly divided into four groups (*n* = 9 per group): (1) normal control group (NC), (2) testosterone-induced BPH model group (TI), (3) EAP group, and (4) prostate antigen supply group (they were sacrificed to obtain antigens for injecting the rats in the EAP group).

The induction of EAP was performed according to Zhou’s [15] method. The nine rats in group 4 were sacrificed by cervical dislocation and the prostate tissues were obtained and homogenized in 0.5% TritonX-100 (Beyotime Biotechnology, Shanghai, China); the homogenates were maintained in an ice water bath. They were then centrifuged at 10,000*g* for 30 min at 4 ℃ and the supernatants were separated. The protein concentrations in the supernatants were assayed by the BCA (bicinchoninic acid) assay (Multisciences Biotechnology Co. Ltd., Hangzhou, China) and diluted to concentrations of 10 mg/ml with 0.1 mol/L PBS buffer (pH 7.2). The nine rats in the EAP group were anesthetized by the intraperitoneal injection of pentobarbital (40 mg/kg). Then, they were intraperitoneally injected with 0.5 ml of Pertussis–Diphtheria–Tetanus vaccine (Wuhan Institute of Biological Products, Wuhan, Hubei, China). Next, a total of 1.0 ml of rat prostate protein extract and FAC (Freund’s Adjuvant Complete; Sigma-Aldrich, MO, USA) emulsion (1:1) was injected subcutaneously into their necks, inner thighs, backs, or hind limbs. The rats in the EAP group were immunized twice, i.e., on day 0 and day 30. The rats in the TI group were injected subcutaneously with 5 mg/kg testosterone propionate (Solarbio Science & Technology Co. Ltd., Beijing, China) (dissolved in corn oil) daily for 45 days. The rats in the NC group did not undergo any treatment. After 45 days, the rats were sacrificed, and the ventral lobes of their prostate glands were quickly removed. One-third of the bilateral ventral lobes were fixed with 4% paraformaldehyde (Beyotime Biotechnology), and the remaining tissues were frozen at − 80 °C.

### H&E staining, Masson’s trichrome staining, hexamine silver staining, immunohistochemical (IHC) staining, and immunofluorescence (IF) staining

The 4% paraformaldehyde-fixed prostate tissue samples from rats in each group were subjected to paraffin embedding and sectioned at a thickness of 5 μm. The tissue sections were subjected to hematoxylin–eosin (H&E) staining, Masson’s trichrome staining, and hexamine silver staining using standard procedures, and examined under a light microscope. IHC staining was performed as previously described [16]. The sections were incubated with primary antibodies against Ki-67 (1:500; Affinity Biosciences, OH, USA) and transforming growth factor β1 (TGF-β1) (1:400; Proteintech Group, Wuhan, China) at 37 °C for 1 h. Then, they were treated with a biotinylated secondary antibody according to the standard protocol. For IF staining, the sections were washed and incubated with antibodies specific for alpha smooth muscle actin (α-SMA) (1:200; Affinity Biosciences) overnight at 4 °C, and then with donkey anti-rabbit IgG (Life Technologies; Thermo Fisher Scientific, Rockford, IL, USA) for 1 h in the dark at room temperature. The slides were mounted by adding DAPI-Fluoromount-G (Southern Biotech) and examined using a fluorescence microscope (Olympus, Tokyo, Japan).

### Grading of the inflammation degree

The prostate samples were graded according to the degree of inflammation: mild, inflammatory foci constituted by 7–14 mononuclear cells; moderate, inflammatory foci constituted by 15–29 mononuclear cells; severe, inflammatory foci constituted by 30 or more mononuclear cells.

### Semi-quantitative assessment of the histopathologic findings in the prostate samples

We used a chart score protocol that was established by Scolnik [17] and then improved by Golomb [18] to perform a better evaluation of the ventral prostate hyperplastic conditions in rats. This protocol considered the acinar morphology, i.e., features such as crowding, intraluminal villosities, loss of basal nuclear polarity, and hyperplastic nodules, which were scored according to their degree of severity and distribution pattern. The examination, description, and scoring of the slides were performed in a blinded manner. Histoscores were expressed in arbitrary units and were obtained from a thorough examination of two H&E-stained sections and one hexamine silver-stained section, considering six different fields of vision for samples from each animal.

### Quantitative real-time PCR (q-PCR)

Total RNA from the samples was extracted using the Trizol reagent (Invitrogen). The purified RNA samples were quantified using a NanoDrop ND-1000 spectrophotometer (NanoDrop Technologies, DE, USA), and 1 μg of each total RNA sample was reverse transcribed using a PrimeScript™ RT Master Mix (TaKaRa, Dalian, China). The cDNA was subjected to quantitative real-time PCR (q-PCR) analysis to detect the mRNA levels using the SYBR reagent kit (TaKaRa) and QuantStudio™ 6 Flex Real-Time PCR System (Applied Biosystems; Thermo Fisher Scientific). All reactions were customized for the specific PCR products and performed in triplicate. The mRNA expression levels of the examined genes were normalized to that of β actin; the relative mRNA expression levels were calculated using the 2^−ΔΔCt^ method.

### Western blotting analysis

The prostatic tissue was ground in liquid nitrogen and the total protein was extracted using a protein extraction kit. Equal amounts (40 μg/lane) of proteins were separated by SDS–polyacrylamide gel electrophoresis; the resultant bands were transferred onto PVDF membranes. After being blocked in 5% bovine serum albumin for 1 h at room temperature, the membranes were incubated with antibodies against TGF-β1 (1:500; Proteintech Group), RhoA (1:5000; Proteintech Group), ROCK1 (1:500; Proteintech Group), and β-actin (1:1000; Affinity Biosciences) overnight at 4 °C. The membranes were washed thrice in TBST for 30 min, and then incubated with horseradish peroxidase-conjugated secondary antibodies (1:5000; Affinity Biosciences) for 1 h followed, by a further washing step for 30 min. Finally, the bands were analyzed using an enhanced chemiluminescence detection system (Pierce; Thermo Fisher Scientific). The data were normalized using β actin as an internal control. All samples were analyzed independently via three repetitions and the mean values were determined.

### Assessment of NO, malondialdehyde (MDA), superoxide dismutase (SOD), and ROS

The SOD activity, NO levels, and MDA levels in the prostate tissues were assessed using a superoxide dismutase (SOD) assay kit (WST-1 method), nitric oxide (NO) assay kit (microwell plate method), and malondialdehyde (MDA) assay kit (TBA method), respectively, according to the manufacturer’s instructions. These commercial assay kits were purchased from the Nanjing Jiancheng Bioengineering Institute (Nanjing, Jiangsu, China). The assays were performed in triplicate, and the total protein concentrations were detected to normalize the data. The reactive oxygen species (ROS) generated in situ in the prostate tissues was assessed using DHE-ROS Assay Kit (BestBio Science, Shanghai, China), according to the manufacturer’s instructions. DAPI was used for staining the nuclei. The stained tissues were visualized using a fluorescence microscope.

### Statistical analysis

The results were analyzed using GraphPad Prism version 7.04 (GraphPad Software, San Diego, CA, USA) and expressed as the means ± standard deviations (SDs). Differences of the mean values between the three groups were analyzed using one-way ANOVA followed by Tukey’s post hoc multiple comparisons test. For all statistical tests, *P* values < 0.05 were considered statistically significant.

## Results

### Effects of the model process on the body weight, prostate weight, and pathological findings in rats

After the completion of the model period, weight-gain was observed in all the groups. Neither hormone nor inflammation significantly affected natural weight-gain. However, the prostate weight increased significantly in the two groups compared with the NC group (Table 1). In addition, we found that the prostates of the EAP group were more prone to bleeding and the prostate capsules partially adhered to the gland, making them hard to remove than the other two groups.

**Table 1:** Effects of the treatments on the prostate and body weights

	Initial body weight (g)	Final body weight (g)	Weight gain (g)	Prostate weight (g)
NC	269.11 ± 12.76	341.11 ± 30.97	72.00 ± 32.57	0.45 ± 0.09
TI	273.11 ± 16.78	362.11 ± 24.82	89.00 ± 34.39	0.82 ± 0.14*
EAP	278.44 ± 15.43	351.78 ± 24.93	73.33 ± 32.01	0.68 ± 0.14*

Values are expressed as the means ± S.D. *Significant difference compared to the NC group, *P *< 0.05

**Fig. 1:**
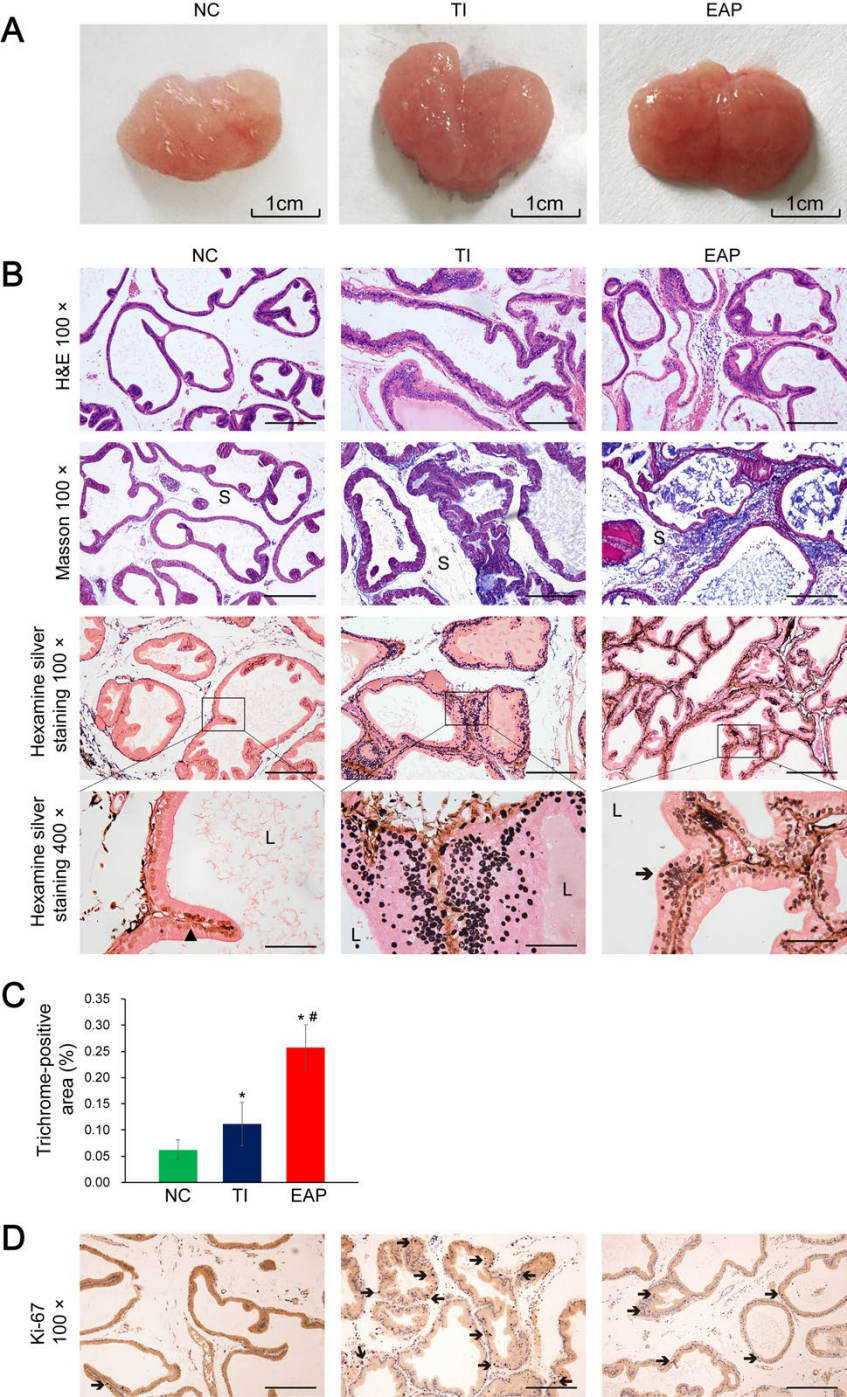
Pathological manifestations in the rat prostate samples. **a** Appearance of the ventral lobes on both sides of the prostate glands. The scale bar in (**a**) is mentioned. **b** H&E staining, Masson’s trichrome staining, and hexamine silver staining of the prostate samples of the rats. The blue colors in the Masson’s trichrome-stained slices indicate collagen fibers. In the hexamine silver-stained slices, the black solid triangular patterns indicate the intact basement membrane and the black arrows indicate the stacking of cells. *S* stroma, *L* lumina. **c** Trichrome-stained areas in the slices (%): the areas positively stained by the Masson’s trichrome stain. **d** IHC staining for detecting Ki-67 in the rat prostate samples. The black arrows indicate Ki-67-positive cells with proliferative potential. Scale bar, 200 μm under 100× magnification and 50 μm under 400× magnification. Each bar in the graph represents the mean ± S.D. *Significant difference compared to the NC group, *P *< 0.05; ^#^significant difference compared to the TI group, *P *< 0.05

The prostate glands of rats from the NC group were mainly characterized by regular acini tapering into low cuboidal cells showing a uniform, monolayered arrangement. Epithelial lesions were found extensively in samples from rats in the TI group, such as extensive high columnar epithelia, multiple layers of disordered epithelial cells, even indications of dysplasia including irregular nuclei and loss of cell polarity, while these lesions were mild and limited in samples from rats in the EAP group (Fig. 1b). Occasionally, intraluminal projections protruding into the acini were found in case of the samples from rats in the NC group. Hexamine silver staining was performed to visualize the basement membrane (Fig. 1b). As the projections had a complete basement membrane, they should be considered as the normal infoldings of the alveolar epithelia. However, the samples from rats in the TI and EAP groups showed greater numbers of projections, and some of these projections had an incomplete basement membrane; thus, they should be considered as pathological manifestations.

In the NC group, the acini were embedded within a thin interstitial stroma. However, the samples of the TI and EAP groups showed increased stromal compartments. Furthermore, Masson’s trichrome staining was performed to evaluate the ECM remodeling, which was indicated by collagen deposition. Compared to the other two groups, inflammation induced collagen deposition strongly in the EAP group (Fig. 1c).

Further, a semi-quantitative assessment of the main histopathologic findings was performed using a score-chart protocol to make the pathological examination complete and uniform. Compared to untreated rats, rats in two model groups had higher cumulative histological scores. And the epithelial lesions in rats under hormonal intervention were more severe than the EAP rats; therefore, they had higher scores (Table 2). Moreover, a large number of ki-67 positive cells were found in the epithelial components of the TI group (Fig. 1d), whereas fewer in the EAP group and NC group.

**Table 2:** Histoscore results for the rats from the NC, TI, and EAP groups

Variable (score values)	NC	TI	EAP
Low power magnification			
* Luminal shape* regular (1), villous (3), papillary (4), cribriform (5)	2.72 ± 0.42	4.50 ± 0.58*	3.44 ± 0.44^#^
* Acinar shape* tubular (1), branched (3), irregular (5)	2.78 ± 0.79	4.72 ± 0.42*	4.17 ± 0.82*
* Interacinar space* large or moderate (1), back-to-back glands (5)	2.11 ± 0.99	2.83 ± 1.03	3.44 ± 1.42*
* Stroma* fine (1), abundant (3), fibrosis/severe SM hyperplasia (5)	1.22 ± 0.25	2.22 ± 0.58*	3.50 ± 0.53*^#^
High-power magnification			
* Epithelial shape* flattened (1), cuboidal (1), cylindrical (3), hexagonal (5)	1.78 ± 0.25	4.67 ± 0.41*	2.56 ± 0.60^#^
* Number of layers* mono(1); oligo, 2–4 (3); pluri, > 5 (5)	1.39 ± 0.21	4.28 ± 0.79*	2.22 ± 0.63^#^
If>1, add: focal(3), diffuse(5)	0.89 ± 0.99	3.83 ± 0.94*	3.44 ± 0.83*
* Alignment *polar (1), apolar (3)	1.83 ± 0.83	2.78 ± 0.25*	2.78 ± 0.34*
If there is piling up of epithelial cells, add 3	1.11 ± 0.52	2.17 ± 1.18*	2.72 ± 0.42*
If there is budding out of epithelial cells into stroma, add 5	0.00 ± 0.00	1.83 ± 1.05*	0.44 ± 0.50^#^
If periacinar clusters of epithelial cells are found, add 3	0.33 ± 0.47	1.44 ± 0.64*	2.56 ± 0.93*^#^
If isolated clusters of epithelial cells are found outside acini, add 5	0.22 ± 0.42	2.00 ± 1.18*	0.94 ± 0.96^#^
* Lesion distribution* (for apolar or budding out cells, no lesion = 0): unilobar: isolated (2), multiple (6). bilobar: isolated (4), multiple (8)	1.72 ± 0.71	7.11 ± 1.66*	6.22 ± 1.99*
* Nuclear shape* round, regular (1); irregular (5)	1.00 ± 0.00	1.83 ± 0.67	1.00 ± 0.00
* Nuclear size* small (2), large (2), small and large in the same acinus (4)	1.11 ± 0.21	2.39 ± 0.46*	1.33 ± 0.53^#^
* Mitoses per field* absent (0); isolated, 1–2 (2); abundant, 3–5 (5); excessive, > 5 (10)	0.00 ± 0.00	2.33 ± 0.41*	0.33 ± 0.62^#^
* Basement membrane* intact (1); interrupted (5)	1.17 ± 0.24	2.56 ± 0.80*	1.22 ± 0.42^#^
Thin (1); thick (5)	1.50 ± 0.41	3.83 ± 0.91*	3.83 ± 0.91*
Total score (arbitrary units)	22.89 ± 1.93	57.33 ± 3.47*	46.17 ± 5.33*^#^

Values are expressed as the means ± S.D. *Significant difference compared to the NC group, *P *< 0.05; ^#^significant difference compared to the TI group, *P *< 0.05

### Significant inflammatory cell infiltration was observed in the samples from rats in the EAP group, with increased expression of multiple cytokines

**Fig. 2:**
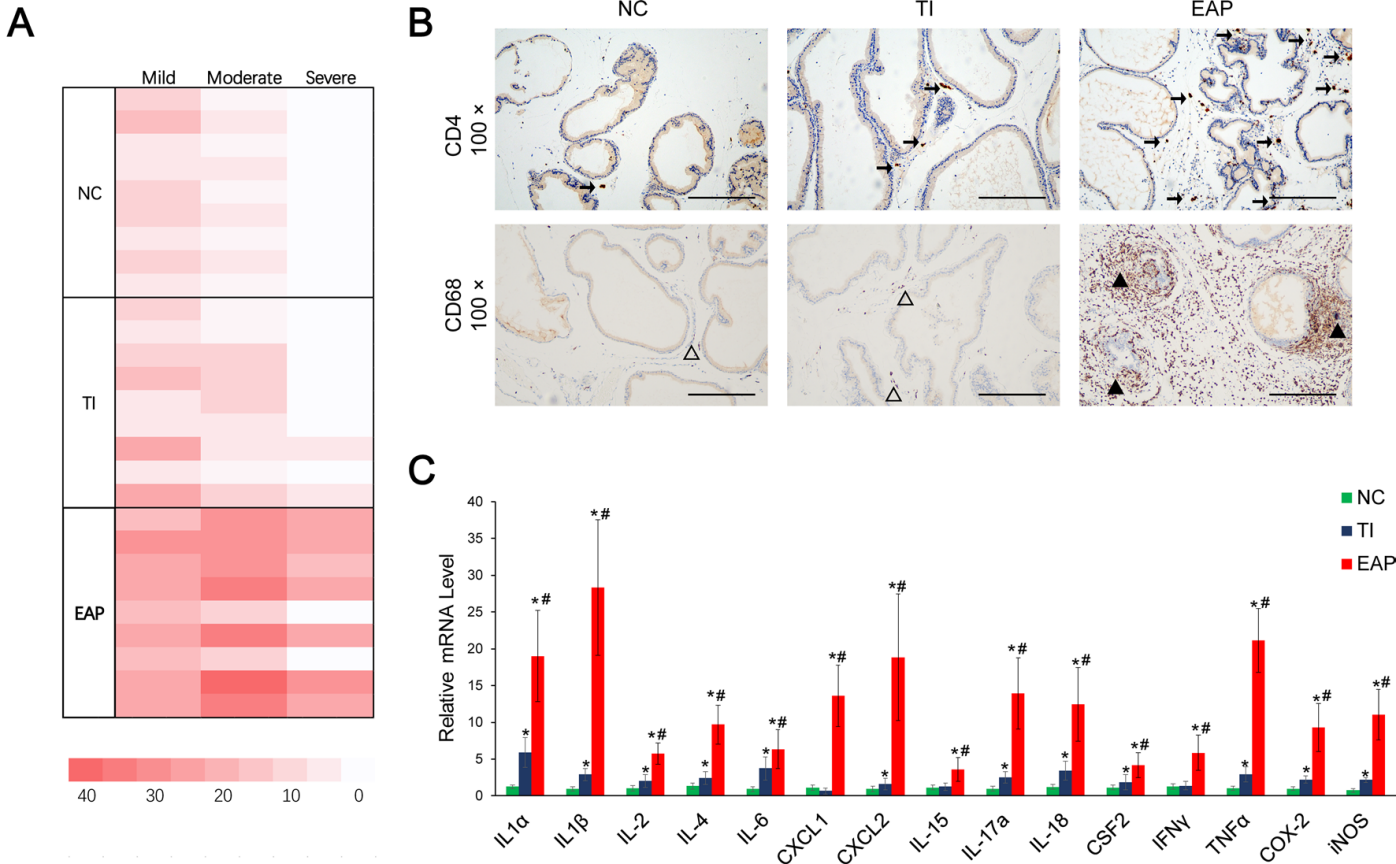
Inflammation degree and cytokine levels in the prostate samples. **a** Inflammation degree and percentage of inflammatory foci/mm^2^ in the prostate sections. **b** IHC staining for detecting CD3 (upper panel) and CD68 (lower panel) in the rat prostate samples was performed to characterize the infiltrated T cells and macrophages, respectively. The black arrow indicates the CD3-positive cells. The open triangles indicate the presence of a small number of infiltrated macrophages, while the black solid triangles indicate the presence of a large number of infiltrated macrophages. **c** The expression of the mRNAs of various cytokine genes relative to that of β actin. Each bar in the graph represents the mean ± S.D. *Significant difference compared to the NC group, *P *< 0.05; ^#^Significant difference compared to the TI group, *P *< 0.05

Based on the HE-stained sections, we analyzed the inflammation degree and percentage of inflammatory foci in the prostate samples from the rats in each group. As the rats of the Wistar strain are sensitive to inflammation [17], there were still some mild inflammatory foci observed in the prostate samples from the rats in the NC group (Fig. 2a). Samples from some rats in the TI group showed increased inflammatory foci, and samples from two of these rats showed severe inflammatory foci. Samples from almost all rats in the EAP group showed obvious inflammatory infiltration, but those from two rats still failed to show a significant degree of inflammation (the subsequent results in this paper no longer include samples from these two rats within the statistical scope of the data for samples from rats in the EAP group). The success rate of our method with regards to inducing a significant inflammatory response was 77% (*n* = 7), suggesting the time for the entire modeling process and the dose of the prostate protein extract still needs to improve. In the prostate glands of most rats from the EAP group, severe inflammatory foci were observed in both the stromal and glandular cavity.

The immunohistochemical analysis for the detection of CD3 and CD68 showed that the proportions of T cells and macrophages in the prostate samples from rats in the EAP group were significantly higher compared to the other two groups (Fig. 2b). Q-PCR was performed to analyze the cytokines potentially involved in the crosstalk between inflammatory cells and prostate cells, and we found that the mRNA levels of most cytokines were significantly elevated in the EAP group, at the same time there were relatively small increases of some cytokines in the TI group (Fig. 2c).

### Reactive stroma and activation of the TGF-β/RhoA/ROCK signaling pathway were observed in the samples from the rats in the EAP group

**Fig. 3:**
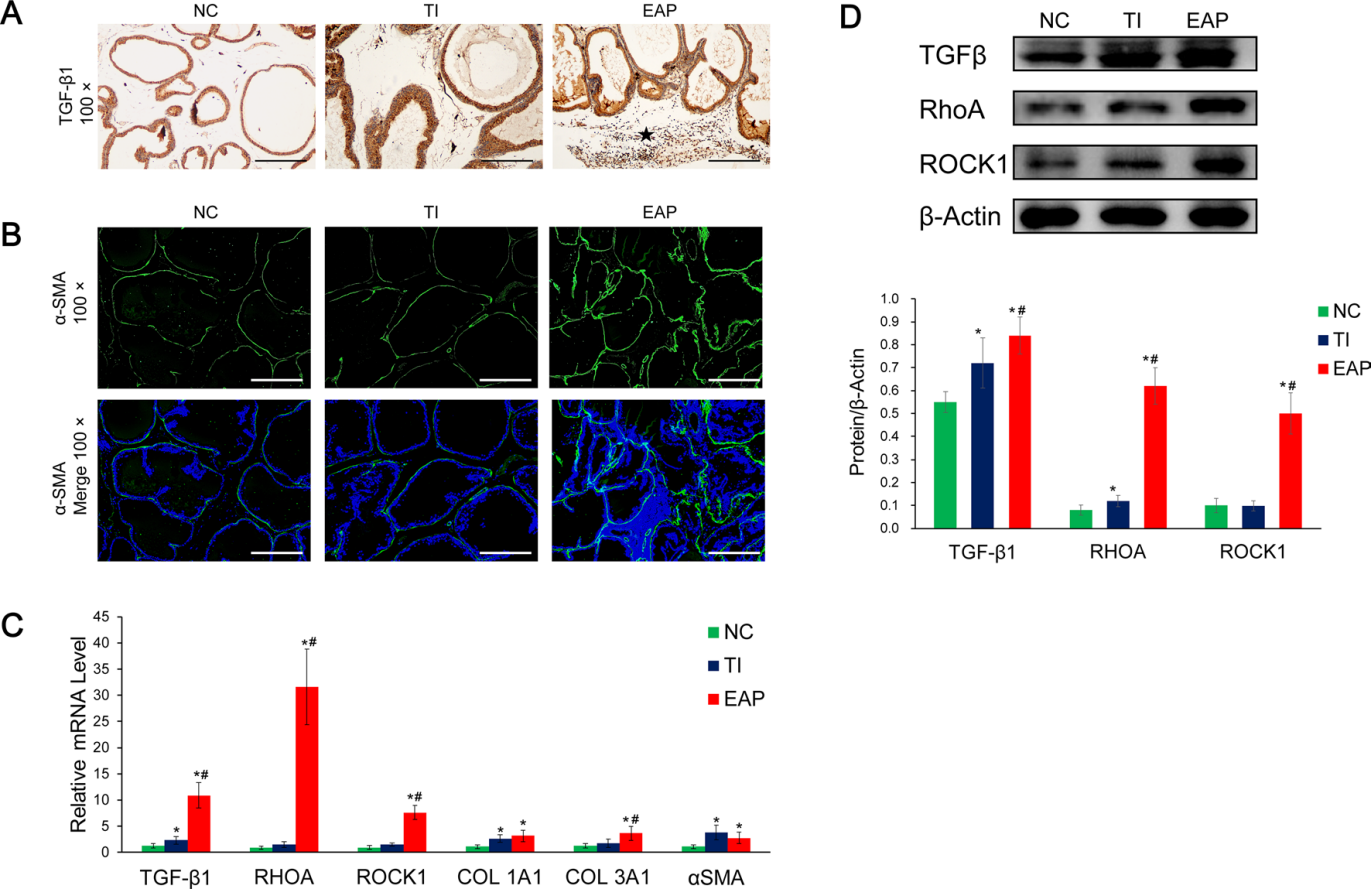
Reactive stroma and levels of the TGF-β/RhoA/ROCK signaling pathway-related components in the prostate samples. **a** IHC staining for detecting TGF-β1 in the rat prostate samples. The black pentagram indicates TGF-β1-positive infiltrating inflammatory cells. **b** Upper panel, IF staining for detecting α-SMA (green) in the rat prostate samples. DAPI staining was performed to detect the nuclei (blue). Lower panel, merging of the α-SMA-stained and nucleus-stained images. **c** The relative mRNA expression levels of the TGF-β1, α-SMA, RhoA, ROCK1, and collagen genes obtained by q-PCR. **d** Representative western blotting results of the TGF-β1, RhoA, and ROCK1 levels in the prostate samples. The graph below indicates the relative densitometric quantification of the proteins. Results are expressed as the ratio of the density of each protein band to the ratio of the density of the β-actin band. Each bar in the graphs represents the mean ± S.D. *Significant difference compared to the NC group, *P *< 0.05; ^#^significant difference compared to the TI group, *P *< 0.05.

The immunofluorescence results showed that the expression of α-SMA in the samples from rats in the EAP group was significantly higher than that in the samples from rats in the other two groups (Fig. 3b), indicating the existence of reactive stroma in the samples from rats in the EAP group. The q-PCR and western blotting analyses results show that the expression levels of TGF-β, RhoA, ROCK1, collagen I, and collagen III in samples from rats in the EAP group were higher than those in samples from rats in the TI and NC groups (Fig. 3c, d), indicating that the RhoA/ROCK signaling pathway played an important role in the formation of reactive matrices in the samples from rats in the EAP group.

### Enhanced oxidative stress, but no obvious oxidative damage was observed in the samples from rats in the EAP group

**Fig. 4:**
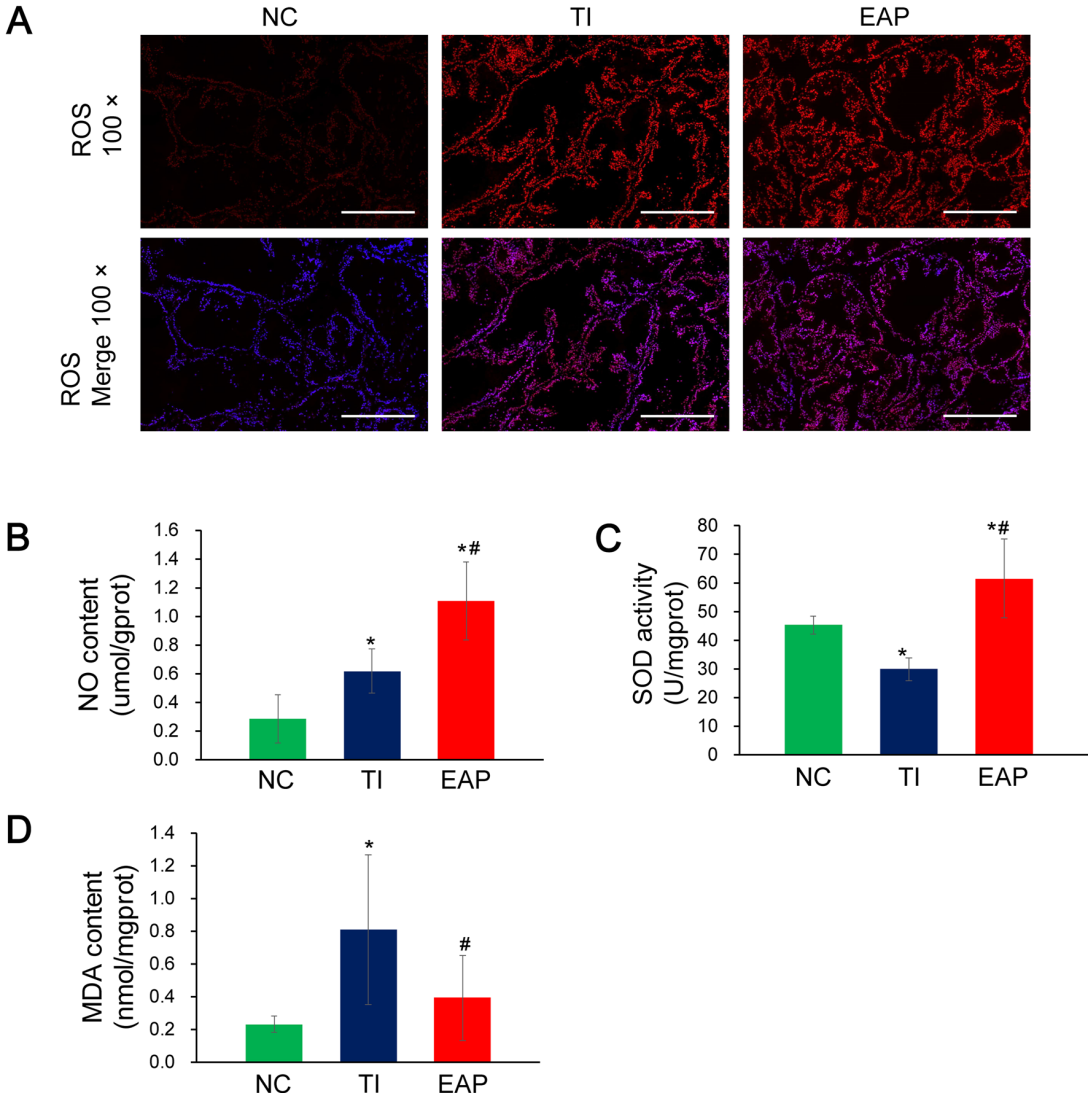
Indicators reflecting the oxidative stress in the prostate samples. **a** ROS produced in situ was assessed using the fluorescent probe DHE. **b** NO concentrations in samples from rats in all the groups. **c** SOD activity in samples from rats in all the groups (assessed using an assay kit: WST-1 method). **d** MDA concentrations in samples from rats in all the groups. Each bar in the graphs represents the mean ± S.D. *Significant difference compared to the NC group, *P *< 0.05; ^#^Significant difference compared to the TI group, *P *< 0.05

The immunofluorescence results showed that the ROS production in the samples from the rats in the EAP and TI groups was significantly higher than that in the samples from the rats in the NC group. The expression levels of inducible nitric oxide synthase (iNOS) and cyclooxygenase 2 (COX-2) (q-PCR results; Fig. 2c), and the NO production levels (ELISA results) in the samples from the rats in the EAP and TI groups were higher than those in the samples from the rats in the NC group. With regards to the antioxidant activity, the SOD activity in the samples from the rats in the EAP group was significantly higher than that in the samples from the rats in the NC and TI groups. With regards to oxidative damage, a lesser amount of MDA, which is a genotoxic lipid peroxide, was generated in the samples from the rats in the EAP group than in those from the rats in the TI group (Fig. 4).

## Discussion

The BPH nodules of individual patients are composed of distinct cell types, and pathological heterogeneity is an underappreciated confounder to identifying novel drug target [19]. Causative molecular pathways involved in a specific BPH phenotype require in-depth research. Our experimental study found that chronic prostatitis induced by autoimmune method in rats showed heterogeneous prostatic hyperplasia from conventional hormone model methods. The EAP rats showed increased stroma compartments and reactive epithelial hyperplasia, while testosterone caused severe epithelial hyperplasia or even atypical hyperplasia.

Many lines of evidence have supported that androgens are permissive, but insufficient for the induction of BPH [20]. The incidence of BPH increases with age in human, but the serum testosterone levels decrease [14]. Androgen supplementation in men does not appear to increase the risk of incident BPH or LUTS (lower urinary tract symptoms) [21, 22]. In the past nearly 50 years, researchers are accustomed to using large doses of testosterone to induce BPH in animal experiments as hormones can quickly and easily induce significant prostate epithelial hyperplasia in young animals. However, it has limitations: (1) the theoretical basis of the hormone-induced model is that the proportion of estrogen and androgen increases with age [20], while disregarding the role of other factors such as inflammation in BPH. (2) Exogenous hormones strongly destroy the hormonal balance of young animals, affecting the immune state of the prostate, as the prostate is also a potential immune organ [23]. (3) Because of the inconsistency of the hormone model induction method, the final pathological status of BPH is not uniform. In particular, there are no consistent reports on whether significant proliferation in the prostatic stroma is observed. For example, some studies reported stromal lesions [11, 12, 24], whereas others only reported epithelial lesions [10, 13, 25–28]. Taken together, the traditional method of hormone-induced model has some limitations in the study of BPH-related mechanism and treatment. Therefore, a new method of induction of hormone-induced model is worthy exploring.

There is already numerous clinical evidence supporting the correlation between BPH and inflammation [5-7]. Many in vitro and in vivo studies have indicated that the activation and dysregulation of prostatic immune cells, and the release of several cytokines and growth factors including IL-2, IL-4, IL-6, IL-17, fibroblast growth factor 2 (FGF-2), were both associated with the initiation and progression of BPH [23]. We found a sharp increase for the size of the stromal compartment in the prostates from EAP rats, accompanied by elevated mRNA levels of several cytokines that stimulated the growth of stromal cells, such as IL-2, IL-4, and IL-18 [23]. Franiel et al. [29] reported that areas of chronic prostatitis tissues had a larger stromal volume than areas of normal prostate tissues. The mean stroma-to-epithelium ratio in the prostate adenomas from men with symptomatic BPH is also higher than that in those from asymptomatic patients [30]. Therefore, inflammation may be a key factor affecting the stromal compartment in both humans and rats, even further promote symptomatic BPH. Unlike rats with chronic prostate inflammation, our research found that large dose of hormone strongly cause epithelial hyperplasia, even atypical hyperplasia in rats. This may be due to the DNA damage induced by serious oxidative stress in the prostate tissues of TI rats. Oxidative stress is commonly observed in hormone-induced BPH models [13, 25, 26], and elevated HIF-1α means significant hypoxia in the model group [24]. However, there was no increase in the MDA levels in the EAP group, because the elevated SOD limited the detrimental effect from ROS. Correspondingly only moderate epithelial hyperplasia was observed in the EAP group. Overall, our results suggested that inflammation and hormones induced heterogeneous BPH in the rat model through different mechanisms.

Recent studies have revealed inflammation implicated as a cause of prostatic fibrosis that contributes to bladder outlet obstruction [31]. Fibrosis in BPH is promoted by the activation of excessive α-SMA-expressing myofibroblasts, which are differentiated from fibroblasts during chronic inflammation and deposit ECM components [32, 33]. TGF-β1 can upregulate the activation of the RhoA/ROCK pathway, promoting cytoskeletal rearrangement and ultimately inducing fibroblast-to-myofibroblast differentiation [34]. In our experiments, elevated TGF-β1 in EAP rats led to activation of RhoA/ROCK pathway and fibrosis of the prostate, while rats under hormonal intervention did not undergo this process.

Autoimmune response, one of the main causes of chronic prostatitis [35-39], is also considered to be as a trigger for the dysregulation of the prostatic immune system and results in the development of BPH nodules [23]. Activated by autoantigens, such as prostate steroid-binding protein, T lymphocytes play a key role as effector and helper cells in the cell-mediated immune response [38, 40]. Besides, prostate cells also released cytokines forming a prostatic network that affect the prostatic proliferation [41]. For example, IL­17 mainly from T lymphocytes, activates the NF­κB pathway, regulates the expression of IL-6, IL-8, and IL-1 in epithelial and stromal cells, and upregulates the expression of COX­2 in macrophages and epithelial cells [23]. COX-2 suppresses the apoptosis by enhancing the expression of anti-death proteins [24]. IL-2, IL-4, and IL­18 stimulate the proliferation of stromal cells. IL-8 induces paracrine of FGF-2 in stromal cells. In our experiment, the inflammatory factor network was examined initially by q-PCR, but further experiments were needed to explore the detailed associations.

Notably, the rats with testosterone administration also showed evidence of inflammatory infiltration compared with the NC group, which might be due to two reasons different from the EAP group. On the one hand, hypoxia and oxidative stress are both observed in hormone-induced BPH rat models and oxidative stress can activate the NF-κB signaling pathway, known as an important inflammatory transcriptional regulator [42]. Abnormal ROS affect the activities of inflammatory mediators and cellular processes involved in the initiation, promotion, and progression of human neoplasms, including BPH and prostate cancer [43]. On the other hand, androgens can be metabolized to estrogens locally within the prostate via the aromatase enzyme [44]. Over-activated aromatase in transgenic mice leads to obstructive voiding [11]. Aromatase inhibitor administered to beagles treated with testosterone and androstenedione, inhibited hormone-induced prostate growth [20]. Testosterone also reduces ER-β and enhances ER-α expression [45], and ER-α stimulation in the prostate results in hyperplasia, inflammation, and dysplasia [20]. In addition, estrogens activate an inflammatory response through the activation of the nonclassical G-protein-coupled estrogen receptor 1, increasing expression of IL-8 [46]. The development of inflammation in response to estrogen is independent of androgens and is the direct response of the prostate to estrogens [44]. Thus, although testosterone alone was administered in our study, the prostates in the TI rats were still affected by endogenous estrogen and developed inflammatory infiltration. Further, the complex metabolism of sex hormones in the prostate makes it difficult to explain the detail mechanism for drug experiment using a hormone-induced BPH model.

In summary, the induction of inflammation can promote the proliferation of prostatic epithelia and stroma, and the effect of inflammation on the stroma is more significant than that of testosterone-induced epithelial changes in rats. In the inflammatory state, increased TGF-β levels contribute to the transdifferentiation of prostate fibroblasts into myofibroblasts, and deposition of new ECM, promoting fibrosis in the prostate gland, in which RhoA/ROCK signaling plays an important role. In study of the therapeutic effects of drugs on stroma components during BPH, we recommend the use of the EAP model, which qualifies as a model of BPH and aids the understanding of the mechanisms underlying inflammation in BPH.
